# Intolerance of uncertainty in adolescents with and without social communication disorder: group and gender differences

**DOI:** 10.3389/fpsyt.2026.1803782

**Published:** 2026-04-15

**Authors:** Alireza Azizpour, Ghorban Hemati Alamdarloo, Setareh Shojaei, Shahram Moradi

**Affiliations:** 1 Special Education Department, School of Education & Psychology, Shiraz University, Shiraz, Iran; 2 Research Group for Disability and Inclusion, Department of Health, Social and Welfare Studies, University of South-Eastern Norway, Porsgrunn, Norway; 3 Research Group for Health Promotion in Settings, Department of Health, Social and Welfare Studies, University of South-Eastern Norway, Tønsberg, Norway

**Keywords:** adolescents, emotional and social development, gender differences, intolerance of uncertainty, social communication disorders

## Abstract

**Objective:**

Intolerance of uncertainty (IU) is a transdiagnostic cognitive-emotional vulnerability associated with anxiety, avoidance, and impaired functioning. Adolescence is a developmental period marked by increasing social and communicative demands, yet little is known about IU in adolescents with social communication disorder (SCD), a condition characterized by persistent difficulties in the pragmatic use of language and social interaction. This study examined group and gender differences in intolerance of uncertainty among adolescents with and without SCD.

**Methods:**

The sample comprised 120 adolescents aged 14–18 years, including 60 adolescents diagnosed with SCD and 60 typically developing peers matched on age, gender, and educational level. Intolerance of uncertainty was assessed using the Intolerance of Uncertainty Scale (IUS). Group and gender differences in overall intolerance of uncertainty and its subscales were examined using two-way analysis of variance and multivariate analysis of variance.

**Results:**

Adolescents with SCD reported significantly higher overall intolerance of uncertainty than adolescents without SCD. Male adolescents also exhibited higher intolerance of uncertainty than female adolescents. These group and gender differences were observed across all four subscales of intolerance of uncertainty, including inability to act, uncertainty being stressful and upsetting, beliefs that unexpected events are negative and should be avoided, and perceptions of uncertainty as unfair. No significant interaction effects between group and gender were found.

**Conclusion:**

Findings suggest that adolescents with SCD experience heightened intolerance of uncertainty, potentially reflecting challenges in managing ambiguity inherent in social communication. Elevated intolerance of uncertainty may represent an important emotional-cognitive vulnerability in this population. Interventions targeting social communication skills and tolerance of ambiguity may help reduce uncertainty-related distress and support psychosocial functioning in adolescents with SCD.

## Introduction

Intolerance of uncertainty (IU) refers to a dispositional tendency to perceive uncertain situations as aversive and to respond to them with negative emotional, cognitive, and behavioral reactions (1, 2). Individuals with high IU experience uncertainty as stressful, threatening, and difficult to manage, which in turn contributes to maladaptive coping strategies such as worry, avoidance, and cognitive rigidity (3, 4). Individuals high in IU also tend to overestimate negative outcomes and rely on avoidance-based coping strategies, which may maintain emotional distress over time (2, 5). IU has been identified as a core transdiagnostic vulnerability factor underlying a range of emotional disorders, including generalized anxiety disorder, social anxiety disorder, obsessive-compulsive disorder, depression, panic disorder, and agoraphobia (6–8). More recent meta-analytic evidence has confirmed robust associations between IU and symptoms across multiple psychological disorders, further supporting its role as a transdiagnostic vulnerability factor (5). In addition, emerging research continues to highlight IU as a key mechanism linked to emotional dysregulation and anxiety-related processes across populations (9, 10).

Theoretical and empirical research suggests that IU contributes directly to excessive worry and indirectly through maladaptive beliefs about worry, negative problem orientation, and cognitive avoidance (4, 11). Individuals with elevated IU tend to interpret ambiguous situations as threatening, show heightened fear responses, and demonstrate reduced tolerance for unpredictability in everyday life (12, 13). Over time, these patterns may lead to increased emotional distress, impaired functioning, and vulnerability to psychopathology (5, 14).

Although most mental disorders emerge during adolescence (15, 16), research on IU has predominantly focused on adult populations. Adolescence is a developmental period characterized by rapid biological, cognitive, and social changes, accompanied by increased exposure to novel, ambiguous, and uncontrollable situations (17). Empirical studies with adolescent samples indicate that higher IU is associated with greater worry, anxiety, and emotional dysregulation (18–20). Nevertheless, IU remains underexplored in specific adolescent subgroups that may be especially vulnerable to uncertainty-related distress.

One such group is adolescents with social communication disorder (SCD). SCD is characterized by persistent difficulties in the social use of verbal and non-verbal communication, including challenges in understanding social cues, interpreting others’ intentions, following conversational rules, and adapting communication to different social contexts (21). These impairments may hinder effective participation in social interactions and increase exposure to ambiguous and unpredictable social situations.

Adolescence may represent a particularly critical period for examining intolerance of uncertainty in individuals with SCD. This developmental stage is characterized by increasing social complexity, greater reliance on peer interactions, and heightened sensitivity to social evaluation (22). At the same time, social communication difficulties associated with SCD may become more pronounced as social demands increase. Research on social communication impairments indicates that difficulties in interpreting social cues, understanding others’ intentions, and navigating peer interactions are associated with increased anxiety and emotional distress in adolescence (23). Such challenges may contribute to heightened sensitivity to ambiguous and unpredictable social situations. The combination of developmental changes and persistent communication difficulties may therefore place adolescents with SCD at increased risk of experiencing uncertainty as stressful, overwhelming, or difficult to manage. Despite these theoretical links, empirical research examining intolerance of uncertainty in adolescents with SCD remains limited.

Social interactions are inherently uncertain, requiring individuals to interpret subtle cues, anticipate others’ responses, and flexibly adjust behavior in real time. Unpredictability in social contexts has been shown to bias the interpretation of ambiguous social cues and increase perceived threat (24). For adolescents with SCD, limitations in pragmatic language and social inference may make such situations particularly challenging, potentially heightening sensitivity to uncertainty and increasing avoidance of ambiguous contexts (25, 26). From this perspective, adolescents with SCD may be especially prone to elevated IU, as uncertainty in social environments may be perceived as overwhelming, uncontrollable, or unfair. This lack of empirical evidence highlights the need for further research examining intolerance of uncertainty in this population.

In addition to group differences, gender differences in IU warrant consideration. Prior research suggests that males and females may differ in social communication skills and emotional processing during adolescence. Females generally demonstrate more advanced language abilities and interpersonal skills, which may facilitate more effective coping with uncertainty in social contexts (27). Empirical research further indicates that gender differences may exist in worry and intolerance of uncertainty during adolescence, with some studies reporting higher levels of maladaptive cognitive responses to uncertainty among males (28). More recent findings also suggest that the relationship between intolerance of uncertainty and emotional or sensory processing may differ by gender, highlighting potential variation in how uncertainty is experienced and regulated (29). However, empirical evidence specifically examining gender differences in IU among adolescents (particularly in clinical or at-risk populations) remains limited, underscoring the relevance of the present study.

Despite growing interest in IU as a transdiagnostic construct, research on intolerance of uncertainty in adolescents with SCD (particularly with respect to potential gender differences) remains limited. Addressing this gap is important for improving understanding of emotional vulnerability in adolescents with communication difficulties and for informing targeted preventive and intervention efforts.

Accordingly, the present study aimed to compare intolerance of uncertainty in adolescents with and without SCD, with particular attention to gender differences. Specifically, this study addressed the following research questions:

1. Are there significant differences in overall intolerance of uncertainty between adolescents with and without SCD, and do these differences vary by gender?
2. Are there significant group and gender differences in the subscales of intolerance of uncertainty?

## Materials and methods

### Participants and procedure

The study population comprised adolescents aged 14–18 years with and without social communication disorder (SCD) residing in the Mashhad-Marghab region of Fars Province, Iran. The final sample consisted of 120 adolescents, including 60 adolescents diagnosed with SCD and 60 adolescents without SCD. The gender distribution was identical across groups, with each group comprising 16 female adolescents and 44 male adolescents. No participants identified as non-binary or other gender categories.

Adolescents with SCD were recruited using purposive sampling. To identify potential participants, all secondary schools in the Mashhad-Marghab region were contacted. School principals and vice-principals were provided with information on the diagnostic features of SCD based on the fifth edition of the *Diagnostic and Statistical Manual of Mental Disorders* (DSM-5; 21) and were asked to refer students who exhibited relevant symptoms.

Adolescents identified through this process were subsequently evaluated by a psychiatrist, and those who met the diagnostic criteria for SCD were included in the study. The diagnosis of social communication disorder (SCD) was established by a licensed psychiatrist, and those who met the DSM-5 diagnostic criteria for SCD were included in the study. The diagnostic assessment involved a comprehensive clinical evaluation, including developmental history, pragmatic language abilities, and social communication functioning. Although a fully structured diagnostic interview, such as the Structured Clinical Interview for DSM Disorders (SCID), was not administered, the assessment was conducted in accordance with standard clinical procedures used in specialized services in the study region.

Adolescents without SCD were recruited using a convenience sampling approach, primarily through schools that agreed to participate in the study. Eligible participants were identified in collaboration with school staff. Information about the study was provided to students, and participation was voluntary. This approach was considered appropriate due to practical and logistical constraints and to facilitate matching with the SCD group on key demographic variables such as age, gender, and grade level in secondary school. No formal clinical screening or structured diagnostic interview (e.g., Structured Clinical Interview for DSM Disorders [SCID]) was conducted for the control group. Furthermore, psychiatric comorbidities were not systematically assessed or used as exclusion criteria in either group. This approach was chosen to reflect the heterogeneity typically observed in real-world adolescent populations.

Comparisons of demographic characteristics indicated no significant differences between the two groups in mean age, educational level, parental employment status, parental divorce or separation, parental death, family size, or family income. These variables were examined using independent-samples t tests or chi-square tests, as appropriate. Thus, the two groups were considered comparable on key sociodemographic characteristics.

### Measures

#### Intolerance of uncertainty scale

Intolerance of uncertainty was assessed using the Intolerance of Uncertainty Scale (IUS; 1, 30). The IUS consists of 27 items designed to measure individuals’ emotional, cognitive, and behavioral reactions to uncertain situations. Items are rated on a five-point Likert-type scale ranging from 1 (not at all characteristic of me) to 5 (entirely characteristic of me). Although the IUS was originally developed and validated in adult populations, research has supported the assessment of intolerance of uncertainty in younger populations. For example, adapted versions of the scale have demonstrated good internal consistency and convergent validity in children and adolescents (31), supporting the relevance of the construct in youth.

The scale comprises four subscales:

1. Inability to act (9 items),
2. Uncertainty being stressful and upsetting (9 items),
3. Unexpected events are negative and should be avoided (5 items), and
4. Uncertainty being unfair (4 items).

Total scores range from 27 to 135, with higher scores indicating greater intolerance of uncertainty. Previous research has demonstrated excellent psychometric properties of the IUS. Freeston et al. (30) reported high internal consistency (α = .91) and good test-retest reliability over a five-week interval (r = .74). Buhr and Dugas (1) similarly reported excellent internal consistency (α = .94) and strong convergent validity through associations with measures of worry and anxiety. Consistent with prior research, the IUS demonstrated excellent internal consistency in the present sample (Cronbach’s α = .96).

In the present study, the paper-and-pencil format of the IUS was administered individually to participants in their schools. Completion of the questionnaire took approximately 30 minutes.

### Ethical considerations

Participation in the study was voluntary. Adolescents provided informed consent prior to participation and were informed about the purpose of the study, the confidentiality of their responses, and their right to withdraw at any time without consequence. All data were treated confidentially and used solely for research purposes. The study protocol was reviewed and approved by the ethical review board of the regional Special Education Organization.

### Statistical analysis

Data were analyzed using descriptive and inferential statistical methods. Means and standard deviations were calculated for total scores and subscale scores of intolerance of uncertainty.

To examine differences in overall intolerance of uncertainty, a two-way analysis of variance (ANOVA) was conducted with group (adolescents with SCD vs. adolescents without SCD) and gender (male vs. female) as independent variables. Prior to conducting the ANOVA, Levene’s test was used to assess the assumption of homogeneity of variances.

To examine group and gender differences across the four subscales of intolerance of uncertainty, a multivariate analysis of variance (MANOVA) was performed. Assumptions of homogeneity of variances and covariance matrices were evaluated using Levene’s test and Box’s M test, respectively. When multivariate effects were significant, follow-up univariate analyses were conducted. Statistical significance was set at *p* <.05.

## Results

**Table 1** presents the means and standard deviations for total intolerance of uncertainty scores by adolescent group (with SCD vs. without SCD) and gender. Overall, adolescents with SCD reported higher levels of intolerance of uncertainty than adolescents without SCD. In addition, male adolescents reported higher intolerance of uncertainty scores than female adolescents across both groups.

**Table 1:** Means and standard deviations of intolerance of uncertainty by group and gender.

Group	Male M (SD)	Female M (SD)	Total M (SD)
Adolescents with SCD	87.41 (18.08)	74.56 (24.31)	83.98 (20.52)
Adolescents without SCD	53.90 (5.01)	47.13 (6.11)	50.52 (6.51)
Total	73.82 (21.84)	56.67 (19.89)	67.25 (22.63)

SCD, Social Communication Disorder; M, mean; SD, standard deviation. Scores represent total scores on the IUS, Intolerance of Uncertainty Scale, with higher scores indicating greater intolerance of uncertainty.

### Group and gender differences in intolerance of uncertainty

A two-way analysis of variance (ANOVA) was conducted to examine the effects of group (adolescents with SCD vs. adolescents without SCD) and gender (male vs. female) on total intolerance of uncertainty scores. Prior to analysis, Levene’s test indicated that the assumption of homogeneity of variances was met.

The ANOVA revealed a significant main effect of group, *F* = 114.68, *p* <.05, indicating that adolescents with SCD exhibited significantly higher intolerance of uncertainty than adolescents without SCD. A significant main effect of gender was also observed, *F* = 11.88, *p* <.05, with male adolescents reporting higher intolerance of uncertainty than female adolescents.

The interaction between group and gender was not significant, indicating that the difference between adolescents with and without SCD did not vary as a function of gender.

**Table 2** presents the means and standard deviations for the four subscales of intolerance of uncertainty (inability to act; uncertainty being stressful and upsetting; unexpected events are negative and should be avoided; uncertainty being unfair) by group and gender. Across all subscales, adolescents with SCD demonstrated higher scores than adolescents without SCD. Similarly, male adolescents consistently reported higher subscale scores than female adolescents.

**Table 2:** Means and standard deviations of intolerance of uncertainty subscales by group and gender.

Subscale	Group	Male M (SD)	Female M (SD)	Total M (SD)
Inability to act	With SCD	28.61 (6.01)	24.06 (7.95)	27.40 (6.82)
	Without SCD	16.87 (3.21)	15.77 (2.54)	16.31 (2.93)
Uncertainty stressful/upsetting	With SCD	28.95 (6.98)	25.13 (9.05)	27.93 (7.70)
	Without SCD	19.10 (2.54)	16.27 (2.38)	17.68 (2.83)
Unexpected events negative	With SCD	16.30 (3.81)	13.69 (4.92)	15.60 (4.25)
	Without SCD	9.67 (2.43)	8.63 (2.08)	9.15 (2.30)
Uncertainty unfair	With SCD	13.55 (3.01)	11.69 (3.84)	13.05 (3.32)
	Without SCD	8.27 (1.70)	6.47 (1.50)	7.37 (1.83)

SCD, Social Communication Disorder; M, mean; SD, standard deviation. Scores represent subscale scores on the IUS, Intolerance of Uncertainty Scale. Higher scores indicate greater intolerance of uncertainty. Subscales include inability to act, uncertainty being stressful and upsetting, unexpected events being negative and should be avoided, and uncertainty being unfair.

### Multivariate analysis of subscale differences

To examine group and gender differences across the four subscales of intolerance of uncertainty, a multivariate analysis of variance (MANOVA) was conducted with group and gender as independent variables and the four subscales as dependent variables. Preliminary assumption testing indicated that the data met the assumptions for MANOVA. Levene’s tests were non-significant for all dependent variables, and Box’s M test indicated homogeneity of covariance matrices.

The MANOVA results showed a significant multivariate main effect of group on the combined dependent variables. Follow-up univariate analyses revealed significant group effects for all four subscales: inability to act (*F* = 102.73, *p* <.05), uncertainty being stressful and upsetting (*F* = 72.82, *p* <.05), unexpected events are negative and should be avoided (*F* = 81.29, p <.05), and uncertainty being unfair (*F* = 110.82, *p* <.05). In all cases, adolescents with SCD reported higher scores than adolescents without SCD.

A significant multivariate main effect of gender was also observed. Univariate analyses indicated significant gender differences across all four subscales, including inability to act (*F* = 8.17, *p* <.05), uncertainty being stressful and upsetting (F = 9.23, p <.05), unexpected events are negative and should be avoided (*F* = 7.90, p <.05), and uncertainty being unfair (*F* = 13.45, *p* <.05). Male adolescents reported higher scores than female adolescents on each subscale. The interaction effects between group and gender were not statistically significant for any of the subscales, indicating that group differences were consistent across genders.

## Discussion

The present study examined intolerance of uncertainty in adolescents with and without SCD, with particular attention to gender differences. Overall, the findings indicated that adolescents with SCD reported significantly higher levels of intolerance of uncertainty than their peers without SCD. In addition, male adolescents exhibited higher intolerance of uncertainty than female adolescents. This pattern is consistent with previous research suggesting that females may demonstrate more adaptive emotional processing and social communication skills during adolescence, which may facilitate more effective coping with uncertain and ambiguous situations (27, 28). Conversely, males may be more prone to maladaptive cognitive responses to uncertainty, including worry and rigidity, which may contribute to higher IU levels. These group and gender differences were observed both for overall intolerance of uncertainty and across all four subscales of the construct.

### Intolerance of uncertainty in adolescents with SCD

Adolescents with SCD demonstrated substantially higher intolerance of uncertainty than adolescents without SCD. This finding is theoretically consistent with the defining features of SCD, which include persistent difficulties in understanding social cues, interpreting others’ intentions, and adapting communication to changing social contexts (21). Social interactions are inherently ambiguous and unpredictable, requiring continuous interpretation and flexible adjustment. For adolescents with SCD, limitations in pragmatic language and social inference may increase the perceived uncertainty of social situations, thereby heightening emotional distress and avoidance.

While the present findings were interpreted primarily in relation to social communication difficulties, alternative mechanisms may also contribute to elevated intolerance of uncertainty in adolescents with SCD. A growing body of research indicates that intolerance of uncertainty is closely linked to social anxiety, worry, and maladaptive cognitive processes, with individuals who have low tolerance for uncertainty reporting higher levels of anxiety in social situations (32, 33). In this context, adolescents with SCD may be particularly susceptible to heightened IU due to repeated challenges in social interactions, which may increase anticipatory anxiety and negative expectations about social outcomes. These processes may contribute to heightened vigilance toward ambiguity and a tendency to interpret uncertain social situations as threatening. In addition, cognitive mechanisms such as rumination and attentional biases may further exacerbate this relationship, as individuals with elevated IU tend to engage in repetitive ruminative thinking that amplifies social anxiety (33).

Evidence from related neurodevelopmental populations also suggests that sensitivity to uncertainty is associated with increased distress and difficulties in social functioning, highlighting the potential role of IU as a mechanism linking social communication challenges and emotional vulnerability (34). Taken together, these findings suggest that elevated intolerance of uncertainty in adolescents with SCD is likely influenced not only by social communication impairments but also by broader cognitive-emotional processes, including anxiety, negative future-oriented thinking, and maladaptive coping strategies.

In addition to social communication difficulties, cognitive and neuropsychological factors may also contribute to elevated intolerance of uncertainty in adolescents with SCD. Difficulties in cognitive flexibility (such as challenges in adapting to changing situations or generating alternative interpretations) may increase reliance on rigid thinking patterns and reduce tolerance for ambiguity (2). Sensory sensitivities, which are commonly reported in populations with communication and neurodevelopmental challenges, may further heighten reactivity to unpredictable environments and contribute to distress in uncertain contexts (35).

From a cognitive-behavioral perspective, individuals with elevated intolerance of uncertainty tend to interpret ambiguous situations as threatening and difficult to manage (12). Adolescents with SCD may be particularly vulnerable to this process, as their communication difficulties can undermine confidence in interpreting social cues and predicting others’ responses. As a result, uncertainty may be experienced as more stressful, unfair, or overwhelming, which aligns with the elevated scores observed across all subscales of intolerance of uncertainty in this group.

### Subscale-specific findings

The finding that adolescents with SCD scored higher on the inability to act subscale suggests that uncertainty may inhibit goal-directed behavior and decision-making in this group. Difficulties in social communication have previously been linked to anxiety, low self-efficacy, and avoidance behaviors (36), which may contribute to hesitation or inaction in uncertain situations.

Similarly, higher scores on the uncertainty being stressful and upsetting subscale indicate that adolescents with SCD experience greater emotional distress in response to ambiguity. Limited social competence and reduced access to effective coping strategies may intensify stress responses when outcomes are unclear (37).

Adolescents with SCD also reported higher levels on the subscale reflecting beliefs that unexpected events are negative and should be avoided. This finding is consistent with research showing that individuals with communication difficulties are more likely to experience anxiety, negative self-concept, and avoidance-oriented coping (36). Avoidance of unexpected situations may function as a short-term strategy to reduce distress but may also restrict opportunities for social learning and adaptation.

Finally, elevated scores on the uncertainty being unfair subscale may reflect perceptions that social demands are unpredictable or biased against the individual. Adolescents with SCD often struggle to adjust communication styles to situational demands, such as modifying language use across contexts or audiences (21). These challenges may foster a sense that uncertainty is imposed externally and experienced as unjust or uncontrollable.

### Gender differences in intolerance of uncertainty

Across both groups, male adolescents reported higher intolerance of uncertainty than female adolescents. This finding is consistent with previous research indicating gender differences in worry and maladaptive cognitive responses to uncertainty during adolescence, with some studies reporting higher levels of such responses among males (28). One possible explanation is that females typically demonstrate more advanced language abilities and interpersonal skills during adolescence, which may facilitate more effective coping with ambiguous social situations (27). Stronger social communication skills may reduce perceived uncertainty by enabling clearer interpretation of social cues and more adaptive responses.

In addition, emerging research suggests that the relationship between intolerance of uncertainty and emotional or sensory sensitivity may differ by gender, indicating variation in how uncertainty is experienced and regulated (29). These differences may contribute to greater sensitivity to uncertainty and less flexible coping strategies among males. Furthermore, male adolescents may experience greater difficulty meeting increasing social demands during adolescence, particularly as peer interactions become more complex and socially evaluative (22). When combined with communication difficulties, these demands may amplify uncertainty-related distress. This interpretation is supported by evidence that social and communicative challenges are more prevalent among males and are associated with higher emotional and behavioral difficulties (38).

The absence of significant interaction effects between group and gender suggests that the association between SCD and intolerance of uncertainty is consistent across males and females. Thus, while gender differences exist, SCD appears to be a robust risk factor for elevated intolerance of uncertainty regardless of gender.

### Implications

The findings of this study have important clinical and educational implications. Elevated intolerance of uncertainty has been linked to anxiety, avoidance, and impaired functioning across developmental stages. Identifying adolescents with SCD who experience high intolerance of uncertainty may help inform early preventive efforts and targeted interventions. Programs aimed at enhancing social communication skills, emotional regulation, and tolerance of ambiguity may reduce uncertainty-related distress and improve psychosocial functioning in this population.

### Limitations and future directions

Several limitations should be acknowledged. First, the study focused exclusively on adolescents aged 14–18 years, which limits the generalizability of the findings to other age groups. Second, the cross-sectional design precludes conclusions about causal relationships between SCD and intolerance of uncertainty. Third, reliance on self-report measures may be subject to response biases. Fourth, psychiatric comorbidities were not systematically assessed or used as exclusion criteria in either the SCD or the control group. As psychiatric conditions rarely occur in isolation, it is possible that undetected comorbidities (such as anxiety or attention-related difficulties) may have influenced levels of intolerance of uncertainty in both groups. Therefore, the observed group differences should be interpreted with caution.

Future research could benefit from longitudinal designs to examine developmental trajectories of intolerance of uncertainty in adolescents with SCD. Qualitative studies may also provide deeper insight into how adolescents with SCD experience and manage uncertainty in everyday social contexts. Additionally, examining the effectiveness of targeted interventions aimed at reducing intolerance of uncertainty in this population represents an important avenue for future work.

## Conclusion

The present study demonstrates that adolescents with social communication disorder exhibit significantly higher intolerance of uncertainty than adolescents without SCD, with this pattern evident across all dimensions of the construct. Male adolescents also reported higher intolerance of uncertainty than females, although the association between SCD and intolerance of uncertainty was consistent across genders. These findings highlight intolerance of uncertainty as an important emotional-cognitive vulnerability in adolescents with SCD, likely reflecting challenges associated with navigating ambiguous and unpredictable social-communicative contexts. Difficulties in interpreting social cues and adapting communication to situational demands may heighten sensitivity to uncertainty, contributing to distress and avoidance in everyday interactions. From a clinical and educational perspective, the results underscore the value of assessing intolerance of uncertainty alongside pragmatic language difficulties and suggest that interventions combining social communication support with strategies aimed at increasing tolerance of uncertainty may help promote psychosocial adjustment in adolescents with SCD. Future research using longitudinal and intervention designs is needed to clarify developmental pathways and evaluate the effectiveness of such integrated approaches.

## Data Availability

The datasets presented in this article are not readily available due to participant confidentiality. Requests to access the datasets should be directed to ghemati@shirazu.ac.ir.
